# Effects of Ultrasonic Treatment on Physical Stability of Lily Juice: Rheological Behavior, Particle Size, and Microstructure

**DOI:** 10.3390/foods13081276

**Published:** 2024-04-21

**Authors:** Si-Hai Han, Jun-Kun Zhu, Lei Shao, Chong-Hui Yue, Pei-Yan Li, Zhou-Ya Bai, Deng-Lin Luo

**Affiliations:** 1 College of Food and Bioengineering, Henan University of Science and Technology, Luoyang 471023, China; 2 Henan Food Raw Material Engineering Technology Research Center, Henan University of Science and Technology, Luoyang 471023, China

**Keywords:** particle size, rheological properties, stability, ultrasonic, lily juice

## Abstract

The aim of this study was to investigate the rheological properties, particle size distribution, color change, and stability of lily juice under different ultrasonic treatment conditions (152 W, 304 W, 456 W, 608 W, and 760 W). The results showed that the lily juice exhibited non-Newtonian shear thinning behavior, and the viscosity decreased with the increase in ultrasonic power. Under ultrasonic treatment conditions, there was no significant change in the pH value and zeta potential value of the samples. The content of cloudy value and total soluble solids (TSS) increased gradually. However, both the sedimentation components and centrifugal sedimentation rate showed a downward trend and an asymptotic behavior. In addition, high-power ultrasound changed the color index (L* value decreased, a* value increased), tissue structure, and particle distribution of the sample, and small particles increased significantly. To sum up, ultrasonic treatment has great potential in improving the physical properties and suspension stability of lily juice.

## 1. Introduction

Changes in food consumption have led to a demand for more nutritious and natural foods, including beverages. Plant-based beverages are a major alternative to milk and for people with dietary restrictions [1] because of the presence of biologically active ingredients with health-promoting properties that help maintain health. At the same time, the rich taste and color greatly satisfy consumers’ dietary needs. Lily is a perennial herbaceous bulbous plant belonging to the family Liliaceae, which is mainly distributed in the central part of Gansu Province, China [2,3]. Lilies can be divided into medicinal lily and edible lily, of which Lanzhou lily is the only sweet lily that can be used as both medicine and food. It is rich in nutrients and functional substances, such as starch, proteins, polysaccharides, vitamins, and colchicine, which have anti-inflammatory, anticancer, antioxidant, anti-aging, and hypoglycemic effects [4,5]. Currently, lilies being processed into beverages is one of the important ways of lily product development. However, problems such as nutrient loss and particle settling during processing and storage of plant beverages pose a great obstacle to product development. Compared with traditional processing technologies (e.g., heat treatment, enzyme treatment, and the use of additives), novel food processing technologies (e.g., high-pressure homogenization, pulsed electric field, ultraviolet irradiation, and ultrasonication) have great potential to improve food quality and enhance food safety [6,7,8,9].

Ultrasonication is considered an emerging and promising technology in the food processing industry because it can produce permanent mechanical, chemical, and biochemical changes in liquids and gases [10]. The main mechanism of ultrasonic action is cavitation, including the formation, growth, and collapse of bubbles [11]. After ultrasonication application, different changes can be observed at the microscopic level of cells and tissues, such as particle surface erosion and cell destruction, size reduction, and changes in molecular conformation. These changes lead to different mechanisms that improve the physical properties of the juice (e.g., consistency, color, turbidity), sensory receptivity [12], and microbial and enzymatic stability [13,14]. For example, in strawberry juice [15], pumpkin juice [16], and guava juice [17], ultrasonication could change the viscosity of the juice and increase turbidity, color index, and bioactive components. The change in physicochemical properties was mainly attributed to the destruction of cell structure and the reduction of particle size [18]. Interestingly, in rheological studies, the viscosity of strawberry juice decreased under the influence of ultrasonication, while the viscosity of pumpkin juice and kiwi juice increased. The viscosity of peach juice showed an increasing trend, followed by a decreasing trend, and then a new increasing trend during ultrasound processing [19]. Ultrasonication has a good retention effect on the natural aromatic components in melon juice, and the enhancement effect of ultrasonication on the flavor of melon juice is better than that of ultra-high-pressure homogenization [20]. Zhu et al. [21] compared four sterilization methods (conventional pasteurization, microwave sterilization, ultrasonic sterilization, and ultra-high-pressure sterilization) and found that ultrasonic sterilization significantly increased the contents of soluble protein, ascorbic acid, soluble pectin, and total soluble solids (TSS) in apple juice. Ultrasonication is also a potential method for controlling the growth of microorganisms in fruit juices and preserving nutritional quality, which could contribute to consumer demand for higher-quality juices [22]. 

As far as non-thermal processing technologies for starch–protein-rich plant juice, high-pressure homogenization technology has been used to improve the physicochemical properties of plant juice [23,24], while the application of ultrasonic technology has been rarely reported. This study explored the influence of different power ultrasonic treatments on the physical properties of lily juice and evaluated the differences in rheological properties, color properties, cloud stability, particle size distribution, potential, and microstructure of lily juice.

## 2. Materials and Methods

### 2.1. Raw Materials and Chemicals

Lanzhou lily (5 kg) was purchased from Dingxi Pavilion (Lanzhou, China). The raw materials were purchased on 6 July 2023 and stored in the refrigerator (4 °C).

### 2.2. Preparation of Lily Juice

The lily bulbs were rinsed with water to remove surface dirt, and the bulbs were boiled in a beaker with distilled water at a high temperature (95–100 °C) for 10 min. To prepare lily juice, the boiled lily petals were mixed with 10 times the mass of water, then grounded with a juicer (HR2838, Philips Home Appliance Co., Ltd., Zhuhai, China) for 1 min and filtered with a single layer of gauze after grinding for 1 min. All the lily juices were placed in 1000 mL sterilized glass bottles and stored in the refrigerator (4 °C) for further ultrasonic treatment.

### 2.3. Ultrasonic Setting

The ultrasonic treatments were performed using an ultrasonic processor (Science-IID, Biotechnology Co., Ltd., Ningbo, China) with a 6 mm diameter probe tip (length 14 cm) and a maximum nominal power of 950 W. The ultrasonic treatment conditions were set as follows: 25 kHz frequency, 10 min (every ultrasonication of 2 s with an interval of 2 s), and powers of 0 W, 152 W, 304 W, 456 W, 608 W, and 760 W. The lily juice sample (150 mL) was placed in a 500 mL glass beaker. The juice sample temperature was kept as low as 25 °C with ice bath during the ultrasonic treatment. All the samples to be further analyzed were stored in glass bottles at 4 °C.

### 2.4. Rheological Properties of Lily Juice

The rheological analysis was performed using a DHR-2 rheometer (Waters Technology Co., Ltd., Shanghai, China) with a flat plate (40 mm in diameter). The lily juice sample of 2 mL was transferred to the surface of the plate. The steady-state rheological tests were carried out with a gap size of 1 mm, a temperature of 25 °C, and the shear rate ranged from 2 to 200 s^−1^ [25]. The shear stress was calculated with Power Law Model Equation (1):

τ = k(γ)^n^ (1)

where τ is the shear stress (Pa), k is the consistency coefficient (Pa.s), γ is the shear rate (s), and n is the flow characteristic index. Three replicates were conducted for each determination.

### 2.5. Particle Size Measurement

The laser particle size analyzer Bttersizer 2600 (Better Instrument Co., Ltd., Dandong, China) was used to measure the particle size and distribution of lily juice. The refractive index value was set to 1.46. The average particle size was calculated by using the volume-weighted average diameter D[4,3] value and the area-weighted average diameter D[3,2] value [26].

### 2.6. Zeta Potential

The zeta potential was measured according to Liu et al. (2016) [27]. All cloudy juice samples were diluted 10 times with deionized water, and the zeta potential at 25 °C was measured with a BeNano 180 Zeta potentiometer (Better Instrument Co., Ltd., Dandong, China). Three replicates were carried out for each determination.

### 2.7. Cloud Stability

To characterize the cloud stability, the lily juice sample 3 mL was centrifuged at 3000 rpm for 10 min. The supernatant was diluted 10 times with deionized water. The absorbance value of the diluted supernatant was measured at 660 nm using a UV-2600 ultraviolet–visible spectrophotometer (Shimadzu Enterprise Management Co., Ltd., Shanghai, China), and the absorbance of deionized water was considered as a blank reference during the spectrophotometric analysis [28].

### 2.8. Color Parameters

The colorimetric analysis was performed according to the method of Bhalerao et al. [29] using an automatic colorimeter SC-80C (Kangguang Instrument Co., Ltd., Beijing, China). The L* (brightness), a* (red–green), and b* (blue–yellow) values of lily juice samples were measured, while distilled water was used as the control. The color variation (∆E) was calculated with Equation (2):

(2) $$∆E=\sqrt{{\left(L^{*}-L_{0}\right)}^{2}+{\left(a^{*}-a_{0}\right)}^{2}+{\left(b^{*}-b_{0}\right)}^{2}}$$

where L_0_, a_0_, and b_0_ are the color values of the untreated beverages, and L*, a*, and b* are those of the treated samples, respectively. Differences in perceivable color can be classified as very different (∆E > 3), different (1.5 < ∆E < 3), and slightly different (∆E < 1.5).

### 2.9. pH and TSS

The pH value of the lily juice sample was measured with a table pH meter (Minyi Electronics Co., Ltd., Shanghai, China). The TSS content was evaluated by a PAL-1 portable digital refractometer (Atago, Tokyo, Japan) at room temperature (25 °C) and presented as average ± difference. The TSS content value was observed by calibrating the refractometer to zero with distilled water and depositing a drop of juice sample on the prism of the refractometer.

### 2.10. Microstructure Observation

The lily juice samples were dispersed on a glass slide and observed by an optical microscope (BA310D, Motic China Group Co., Ltd., Xiamen, China) with a digital camera. The microstructure images were taken at 100× magnification. 

### 2.11. Stability Assessment of the Ultrasonicated Juice

The treated sample was immediately stirred and poured into the sterilized glass bottle and tightly closed. In order to determine the stability, the ultrasonicated juice samples were stored in upright 50 mL sealed glass bottles for 0 h, 48 h, and 96 h, respectively, at room temperature (25 °C) [30]. The accelerated aging method by centrifugal sedimentation [31] was carried out for the stability assessment of the ultrasonicated juice. The juice sample of 8 mL in a calibrated centrifuge tube was centrifuged for 10 min at 3000 rpm with a centrifuge (TG16-WS, Xiangyi Laboratory Instrument Development Co., Ltd., Hunan, China). Following that, the sediment was weighed, and the index of centrifugal sedimentation was expressed as Equation (3):

(3) $$centrifugal\;sedimentation\;value(\%)=\frac{m_{0}}{m}$$

where m_0_ is the mass of the sediment after centrifugation, and m is the total mass of the sample. Three replicates were set for each experiment.

### 2.12. Statistical Analysis

All experiments were performed in triplicate, and the data were subjected to one-way analysis of variance (ANOVA) using SPSS 22.0. The results were expressed as mean ± standard deviation (SD), and the statistical significance was set to *p* < 0.05.

## 3. Results and Discussion

### 3.1. Steady-State Rheological Properties

**Figure 1:**
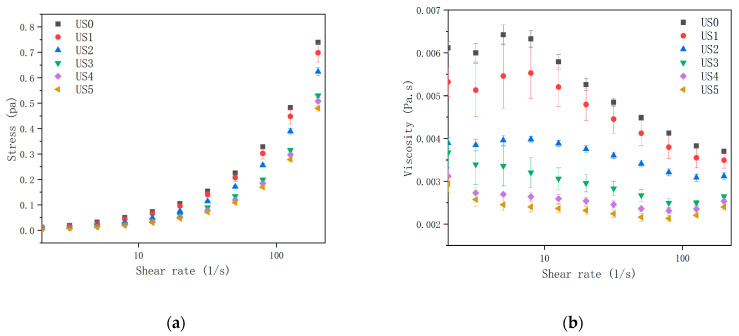
Steady-state rheological curves of lily juice at different ultrasonic power levels. Curve of shear stress with shear rate (**a**) and curve of apparent viscosity with shear rate (**b**). Three independent measurements were made for each sample, with the results expressed as the mean and the error bar as the standard deviation. US0: no ultrasonic treatment; US1: 152 W; US2: 304 W; US3: 456 W; US4: 608 W; US5: 760 W.

The rheological properties of food are of great significance for unit operation design, process optimization, and high-quality product assurance. Figure 1a shows the influence of ultrasonic power and shear rate on the shear stress of lily juice. The shear stress of ultrasonic-treated juice was lower than the control, and the difference was magnified with the increase in shear rate. Table 1 shows the fitting of shear stress with the shear rate curve by a power law model, and it can be seen from the table that all regression coefficients were above 0.996, indicating that the power law model could describe the rheological curve of samples well. The consistency index K of the control was the highest, and the K value decreased from 0.00739 to 0.00253 with the increase in ultrasonic power, while the value of n increased from 0.868 to 0.975, which was similar to the result of spinach juice treated by high-intensity ultrasonication [32]. The decrease in K value indicated that all the viscosity of the ultrasonic treated samples decreased, which conformed to the trend in Figure 1b. The viscosity of the control juice was 0.00612 Pa.s. When the ultrasonic power reached 760 w, the viscosity dropped to 0.00019 Pa.s. The shear thinning behavior (*n* < 1) indicated that the lily juice samples belonged to non-Newtonian fluids. 

The rheological properties of products were largely dependent on the mean particle size, particle size distribution (PSD), particle shape, and the way particles interact with particles and with serum phases. The reduction in particle size could contribute to greater interactions, resulting in an extensive network in tomato juice [33], while the smaller particles could reduce flow resistance, resulting in inconsistent pineapple pulp [34]. The increase in n and the decrease in K could be explained by the destruction of the suspended solids in the juice, resulting from the decrease in particle size. The lubricant effect of smaller size particles and smooth surface morphology also leads to a decrease in flow resistance [35]. 

Temperature, particle size, and soluble solids also had considerable influence on its rheological properties [36]. It was reported that decreasing the size of pectin molecules could reduce the viscosity [37] and lead to increased flow behavior [38]. The depolymerization of cell wall pectin could also lead to a decrease in the viscosity [39].

### 3.2. Particle Size Analysis

**Figure 2:**
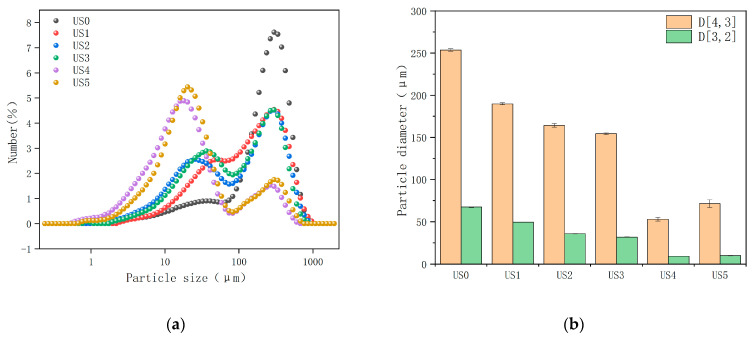
Particle size distribution curve (**a**) and average particle size (**b**) of lily juice under different ultrasonic power. D[4,3]: volume mean diameter; D[3,2]: area mean diameter D[3,2]. US0: no ultrasonic treatment; US1: 152 W; US2: 304 W; US3: 456 W; US4: 608 W; US5: 760 W.

The particle size distribution and average particle size (D[4,3] and D[3,2]) of the lily juice samples were shown in Figure 2a,b, respectively. An obvious large-size particle peak was observed in the control sample, with the particle size distribution ranging from 82.36–677.55 μm. The ultrasonic power above 304 W led to an increased interval of smaller particles, and a bimodal distribution of particles was observed (Figure 2a). With the increase in ultrasonic power, the peak of small particles gradually increased and shifted to the left. When the ultrasonic power reached 608 W, the peak of small particles was significantly higher than that of large particles, and the particle size distribution was mostly in the range of 3.1–51.55 μm. The presence of a single peak was due to less stable sample components clumping together to form macromolecular polymers so that only one peak represents the polymer peak [40]. The multiple peaks were separate distributions of multiple components. This phenomenon was also observed during high-pressure homogenization [41]. The particle size distribution was due to the fact that the large aggregate was broken by ultrasonic action, forming small particles. When the ultrasonic power increased, the particles were damaged more, the particle size range was wider, and the large particle peak was more shifted to the small particle peak. 

The values of D[4,3] and D[3,2] decreased with the increase in ultrasonic power (Figure 2b), indicating that the two size particles had similar effects in the ultrasonic process. The values of D[4,3] and D[3,2] were affected by large and small-sized particles [42], respectively. The decrease in particle size could be attributed to cavitation, shear, and turbulence caused by ultrasonic treatment, resulting in the effective destruction of most particles [43]. For example, in the cavitation process, the formation and rupture of bubbles would produce high temperatures, high pressures, and strong microjets and shock waves, which could cause serious damage to particles and droplets in the liquid. The instantaneous temperature was conducive to the decrease in viscosity and the increase in cavitation bubbles, resulting in a more violent collapse and enhanced destructive effect [44]. The increase in the number of small particles was also associated with the formation of more debris from the cell membrane and cell wall [35]. In our study, the average particle size also showed a partial progressive behavior, possibly because the force required to destroy smaller particles would become larger, and only by further increasing the power could small particles be broken, as reported by Leite et al. [45]. The temperature increase could induce protein aggregation, leading to a slight increase in particle size [46], which may be caused by transient high temperatures in the local environment during ultrasonication.

The large particles were more likely to flocculate than small particles, and the reduced particle size was conducive to reducing the van der Waals forces between particles [47]. Meanwhile, according to Stokes’ law, the particle settling velocity was proportional to the particle diameter and inversely proportional to the viscosity of the dispersion medium. The smaller particles were beneficial to slow down gravity separation and aggregation, thus improving the physical stability of the turbid juice system.

### 3.3. Zeta Potential

**Figure 3:**
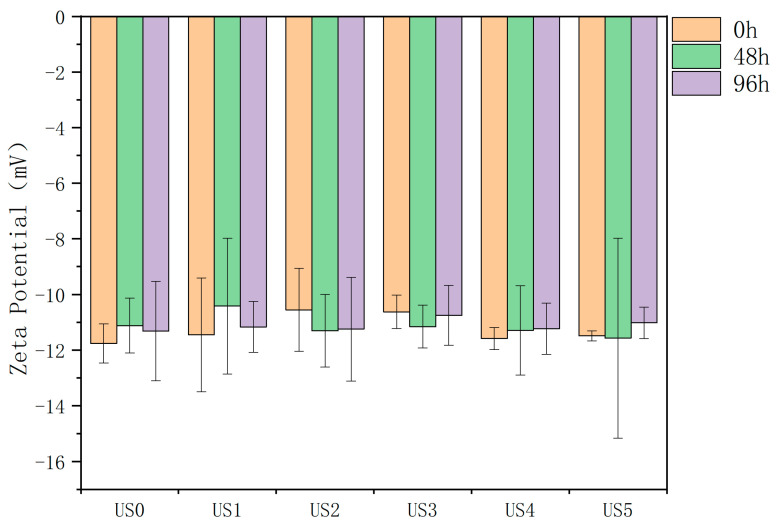
Changes of zeta potential of lily juice under different ultrasonic power and storage conditions. US0: no ultrasonic treatment; US1: 152 W; US2: 304 W; US3: 456 W; US4: 608 W; US5: 760 W.

Zeta potential could be used to analyze the potential stability of the system. The charged state on the surface of particles in the lily juice system before and after ultrasonic treatment is shown in Figure 3. The zeta values of all samples were negative, indicating that there were more negatively charged particles than positively charged particles in the system. This was similar to the charged state of most fruit and vegetable juices. The negative electric state was related to the isoelectric point of protein in the lily juice [48]. The study found that when the environmental pH value of the system was higher than the isoelectric point of protein in the system, the solution showed a negative electric charge, and vice versa. The ultrasonic treatment did not change the pH value of the system, and the pH value was close to neutral at about 6.7, which was higher than the isoelectric point of protein (Table 2). When the particles were surrounded by a negatively charged pectin protective layer, it could also result in the overall surface being negatively charged [49].

As shown in Figure 3, the zeta potential value of the control group is −11.76 ± 0.71 mV, while in previous research reports, the absolute value of the zeta potential of the system is at least ±25 mV to provide enough repulsive force to overcome the attraction between particles [50]. This indicated that the sample system is in an unstable state from the perspective of zeta potential analysis. The ultrasonic treatment did not significantly change the zeta potential value of the system, which was similar to the research results in Huyou juice [51], which also indicated that the particle charge and electrostatic repulsion were not the main reasons for the stability changing of lily juice. The unchanged pH value of the sample may be one of the reasons for the unchanged zeta potential value [52]. In this study, the zeta potential value of the samples during the storage period also did not change significantly. Combined with the sedimentation experiment, the zeta potential value of the current system could not effectively inhibit the aggregation and sedimentation of particles.

### 3.4. Color Value

Appearance is the first factor considered by consumers in the process of deciding to accept or reject the observed product, and color is the primary appearance attribute [53]. As shown in Table 2, the L* value of the control (20.27 ± 0.07) was the largest. With the increase in ultrasonic power, the L* value gradually decreased significantly, while the a* value showed the opposite trend, and the b* value did not change significantly, indicating that the brightness of the juice decreased and the redness increased with the increase in ultrasonic power. The color change was directly related to the browning reaction during processing [54]. The contents of polyphenols and ascorbic acid in lily were high, and browning was easy to occur during processing and storage. The cavitation helped to reduce the oxygen content in the juice [19,55]. However, the high amount of carbohydrates (e.g., starch, fiber) and proteins in lilies, accompanied by the products of the Maillard reaction formed by the heat effect produced during processing, also affected the color change [44]. Cao et al. [56] attributed the decrease in the L* value of bayberry juice to oxidative darkening, which was caused by free radicals generated during the cavitation process. The structural changes caused by the ultrasonic process could promote the further release of bioactive compounds and minerals, such as carotenoids and vitamins in plant cells, which were more likely to undergo free radical oxidation reactions during the cavitation process [57], thus exacerbating the color change. The improvement of color properties (a* and b*) is related to the release of carotenoids and anthocyanins, which is caused by the destruction of juice tissue under the condition of high-intensity ultrasonication [22]. The enhancement of red color was also related to the isomerization of carotenoid compounds [58].

The L* value was highly correlated with the change in turbidity of the system, mainly because ultrasonic homogenization changed the size and distribution of particles. The higher turbidity resulted in lower clarity or darker color of the juice [28]. The decrease in the L* value corresponds to the increase in the turbidity value. The total color difference ∆E represents the color change of turbid juice. The ∆E was less than 2 when the ultrasonic power was below 456 W, and the color difference at this time could not be distinguished by the naked eye. When the power continued to increase, the ultrasonic treatment resulted in higher color changes.

### 3.5. pH and TSS

As shown in Table 2, the ultrasonic treatment did not change the pH value (6.66–6.72) of the lily juice system, which was similar to the research results of bayberry juice [56] and guava juice [59]. This result demonstrated that ultrasonic treatment possessed the advantage of maintaining the pH of the juice.

TSS represents the total soluble solids dissolved in water, including sugars, proteins, and free acids. The TSS content of lily juice was increased by ultrasonic treatment (Table 2), which was due to cell rupture caused by cavitation, resulting in intracellular substances, such as sugars (glucose, fructose, and sucrose), being released into the medium [21]. Increasing the ultrasonic power also accelerated the diffusion of sugar, resulting in higher dry matter content and sugar content in the juice. However, TSS decreased significantly in the ultrasonic treatment of strawberry juice [22], which may be related to the conversion of organic acids to other molecules or sugars. Ultrasonication did not change the TSS content in bayberry juice, indicating that the change in TSS was also closely related to the substances contained in the system itself.

### 3.6. Cloud Value

**Figure 4:**
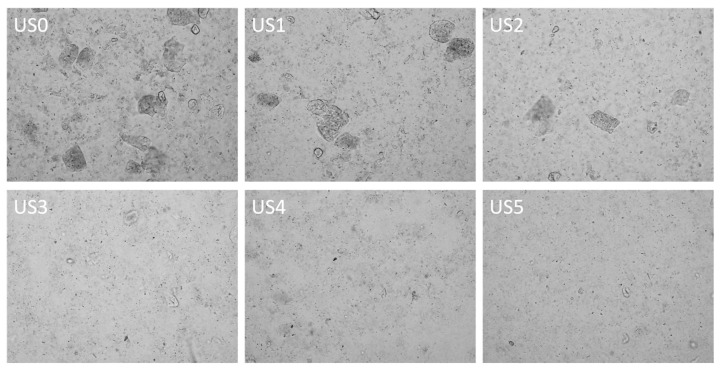
Optical microscope observation of lily juice under different ultrasonic power (optical microscope 100×). US0: no ultrasonic treatment; US1: 152 W; US2: 304 W; US3: 456 W; US4: 608 W; US5: 760 W.

The cloud is a complex mixture of starch, protein, pectin, lipids, hemicellulose, cellulose, and other minor components. In orange juice [37], the loss of cloud leads to a decrease in consumer acceptability. The cloud particles could give the juice a characteristic flavor, color, and texture. The changes in cloud value could be used to predict the stability of juice. The higher cloud value indicated better stability. As shown in Table 2, the cloud value gradually increased with the increase in ultrasonic power, which is consistent with the phenomenon of strawberry juice [22], guava juice [17], and peach juice [19]. The suspended particles (plant cells, fibers, carbohydrates, and proteins) in the juice were broken by ultrasonication through acoustic cavitation, and small-sized particles did not precipitate during the centrifugation process. The increased fine particles enhanced the absorbance value and cloud value. The particle size distribution (Figure 2) and microscope observation (Figure 4) also demonstrated that ultrasonication increased the number of small particles. The weakening of enzymatic reactions in the system could also lead to the change in turbidity value. During the cavitation process, the ultrasonic with a high shear effect could denature the pectin methyl esterase (PME) molecules and reduce the degradation of pectin [59]. This also helps to improve the turbidity of the juice. In addition, cavitation induces the rupture of tissue cells, leading to the release of intracellular compounds (e.g., carotenoids and sugars), which also increases the cloudiness of the juice [18].

### 3.7. Optical Microstructure

The microscopic characteristics of ultrasonic-treated lily juice were observed with an optical microscope. The control juice had many individual cells, cell clusters, and other microscopic particles (such as starch, protein, pectin, and fiber), indicating that lily juice was a complex system (Figure 4, US0). With the increase in ultrasonic power from 152 W to 304 W, the larger particles in the sample (cell clusters or other aggregated particles) gradually decreased, and the small particles (mainly separated starch, protein, cell fragments, etc.) increased significantly (Figure 4, US1–US2). This result was similar to that of guava juice [17] and pumpkin juice [16]. The erosion of particles was caused by the physical effects (shear forces and cavitation) generated during the ultrasonic process. It could be deduced that the intracellular substances were released into the lily juice when the cells were destroyed, which was one important reason for the change in the physical and chemical properties of the juice. When the ultrasonic power reached above 456 W, few large-sized particles existed, and the distribution of small particles became more uniform and denser. This is mainly because the breakdown of the material shifted from cell clusters and aggregated particles to smaller particles, such as separated starch, protein particles, and cell fragments, with the ultrasonic power increase. The microscopic characteristics were consistent with the particle size analysis (Figure 2). The change in the structure of small particles was also an important factor affecting the flow of liquid.

### 3.8. Stability Assessment

**Figure 5:**
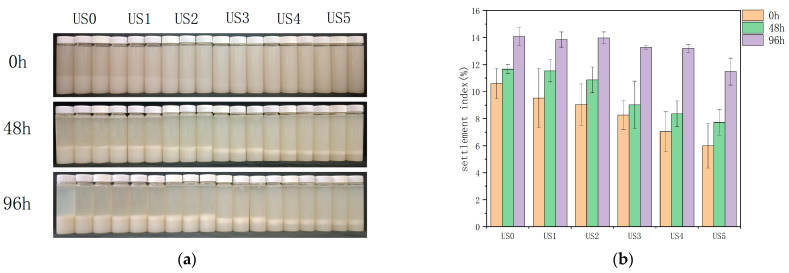
The settlement experiment (**a**) and centrifugal precipitation rate (**b**). US0: no ultrasonic treatment; US1: 152 W; US2: 304 W; US3: 456 W; US4: 608 W; US5: 760 W.

The sedimentation experiments allowed a direct, accurate, and objective assessment of the stability of the juice system. At the beginning of storage (0 h), sedimentation began to appear at the bottom of the bottle while the juice still remained cloudy, which was the downward sedimentation of the larger particles in the system by gravity when the force to maintain the particles in suspension was lower than the gravitational force on the particles. With increasing storage time (48–96 h), the small particles in juice gradually flocculated and precipitated under the action of surface (electrostatic, van der Waals) and mass (gravity, centrifugal) forces, resulting in a relatively transparent aqueous phase in the upper layer (Figure 5a).

The deposition of the lily juice after ultrasonication was less and showed a certain degree of gradualness compared with the control. The possible reason was that there were more large particles in the control group, and ultrasonication reduced the particle size so that small particles predominated. The small-sized particles were less likely to settle by gravity, and the stability of the juice was improved. The centrifugal precipitation result showed that ultrasonication could effectively reduce the centrifugal sedimentation rate and increase the turbidity of lily juice (Figure 5b), which was consistent with the settlement experiment.

The samples subjected to high-power sonication were more stable with less sedimentation compared to the control. The sedimentation rate decreased with increasing ultrasonic power. At 608 W, the decrease in sedimentation rate tended to stabilize, indicating that 608 W could be the optimum ultrasonic power.

## 4. Conclusions

The effects of different ultrasonic power conditions (152~760 W) on rheological properties, microstructure, and sedimentation indexes of lily juice were investigated. The ultrasonic destroyed the structure of lily juice and reduced the particle size of lily juice. These changes also led to other physical properties, such as increased TSS and turbidity, decreased viscosity and brightness, and delayed sediment formation, improving the suspension stability of cloudy juice. However, the pH value and zeta potential value of lily juice did not change after ultrasonication, which is also noteworthy. The above results showed that the physical properties of lily juice could be improved by ultrasonication, which provided some reference for the research and production of lily juice.

## Figures and Tables

**Table 1:** Influence of ultrasonication on rheological behavior of lily Juice (fitted with the power law model).

	US0	US1	US2	US3	US4	US5
K	0.00739 ± 3.27318 × 10^−4 a^	0.0061 ± 2.56015 × 10^−4 b^	0.00442 ± 1.33271 × 10^−4 c^	0.00338 ± 2.70179 × 10^−4 d^	0.00282 ± 9.7261 × 10^−5 e^	0.00253 ± 1.49411 × 10^−4 e^
n	0.86803 ± 0.0097 ^a^	0.89714 ± 0.01097 ^b^	0.93504 ± 0.00899 ^c^	0.94858 ± 0.01648 ^cd^	0.96742 ± 0.00921d ^e^	0.97564 ± 0.01424 ^e^
R^2^	0.998	0.996	0.998	0.998	0.997	0.996

Different superscript letters in the same line (a–e) indicate significant differences (*p* < 0.05). In the table, US0: no ultrasonic treatment; US1: 152 W; US2: 304 W; US3: 456 W; US4: 608 W; US5: 760 W.

**Table 2:** Other physicochemical and physical properties of lily juice.

	L*	a*	b*	∆E	TSS	pH	Cloud Value
US0	20.27 ± 0.07 ^a^	2.9 ± 0.19 ^a^	5.87 ± 0.25 ^a^	0	1.87 ± 0.06 ^a^	6.72 ± 0.03 ^a^	0.19 ± 0.002 ^a^
US1	19.90 ± 0.07 ^ab^	2.95 ± 0.05 ^a^	6.84 ± 0.82 ^a^	1.18 ± 0.77 ^b^	1.9 ± 0.1 ^ab^	6.7 ± 0.02 ^a^	0.196 ± 0.001 ^a^
US2	19.84 ± 0.4 ^b^	4.15 ± 0.16 ^b^	6.94 ± 1.1 ^a^	1.85 ± 0.32 ^bc^	1.93 ± 0.06 ^ab^	6.66 ± 0.04 ^a^	0.219 ± 0.008 ^b^
US3	19.86 ± 0.12 ^ab^	5.14 ± 0.08 ^c^	6.02 ± 0.32 ^a^	2.32 ± 0.2 ^cd^	2.03 ± 0.06 ^bc^	6.66 ± 0.03 ^a^	0.244 ± 0.002 ^c^
US4	19.79 ± 0.25 ^b^	5.29 ± 0.44 ^c^	6.04 ± 0.36 ^a^	2.46 ± 0.65 ^cd^	2.1 ± 0.1 ^cd^	6.68 ± 0.02 ^a^	0.26 ± 0.003 ^d^
US5	19.71 ± 0.07 ^b^	5.98 ± 0.31 ^d^	5.94 ± 0.26 ^a^	3.14 ± 0.39 ^d^	2.2 ± 0.1 ^e^	6.72 ± 0.03 ^a^	0.283 ± 0.003 ^e^

TSS: Total soluble solids (Brix). Three independent measurements were performed for each sample, and the results are indicated as the mean ± standard deviation. Different superscript letters (a–e) in the same column indicate significant differences (*p* < 0.05). US0: no ultrasonic treatment; US1: 152 W; US2: 304 W; US3: 456 W; US4: 608 W; US5: 760 W.

## Data Availability

The original contributions presented in the study are included in the article, further inquiries can be directed to the corresponding author.
